# Supplementary material for: Global mapping of CARM1 substrates defines enzyme specificity and substrate recognition
Source: Nat Commun. 2017 May 24;8:15571. doi: 10.1038/ncomms15571 (PMC5458078; doi:10.1038/ncomms15571)
Supplement: Supplementary Information — Supplementary Figures and Supplementary Tables [file ncomms15571-s1.pdf]

Supplementary Figure 1.

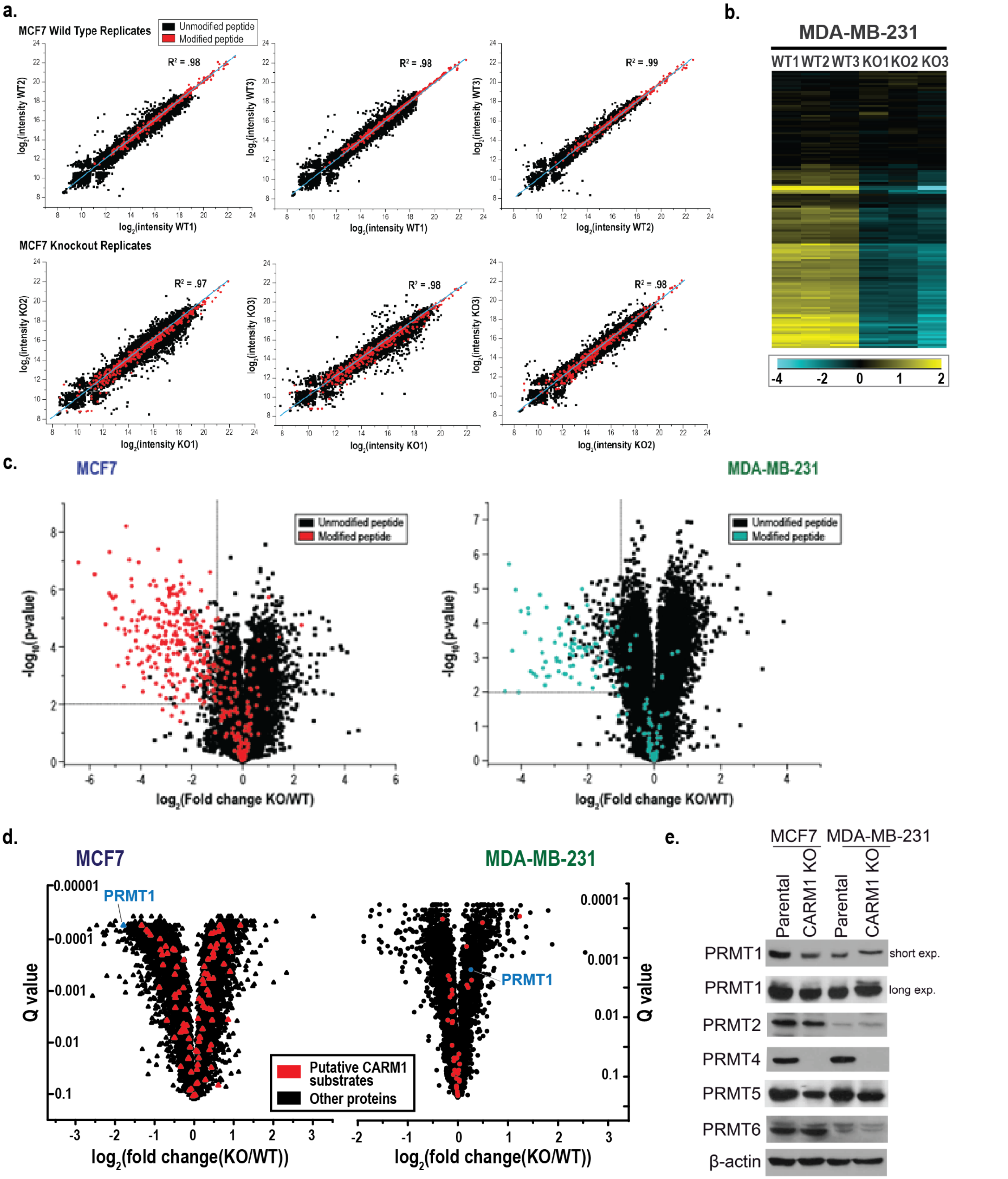

Supplementary Figure 1. Global profiling of CARM1 substrates using quantitative mass spectrometry.

- **a.** Reproducibility between three biological replicas achieved in experiments using MCF7 cells. The indicated Pearson correlation R^2 values include both modified and unmodified peptides.
- **b.** Identification and quantification of ADMA peptides affected by CARM1 loss in MDA-MB-231 breast cancer cell line using three biological replicas of wild type (WT) and knockout (KO) cells. The heat map displays hierarchal clustering using Pearson correlation of mean normalized $\log_2$ transformed intensities of ADMA peptides.
- **c.** Volcano plots demonstrating changes in abundance of both modified and unmodified peptides in MCF7 (*left*) and MDA-MB-231 cells (*right*) using three biological replicas of each cell line. A large subset of modified peptides in each cell line (~50%) exhibited extreme reduction in abundance, meanwhile the abundance of a very few unmodified peptides (~1%) was altered upon CARM1 deletion (p value <0.01; two-tailed Student's t test).
- **d.** Volcano plots demonstrating changes in overall protein abundances in MCF7 (*left*) and MDA-MB-231 cells (*right*) using three biological replicas of each cell line. With six exceptions, no significant changes below Q-value of 0.01 (FDR of 1%; Storey correction for multiple hypothesis testing) in protein abundance (± 2-fold) were measured for any putative substrates (denoted in red), suggesting that the observed decrease in the abundances of ADMA-containing peptides was not due to the decrease in abundance of the corresponding proteins. PRMT1 (denoted in blue) was detected with 2.5 fold decrease in MCF7 but not in MDA-MB-231 cells.
- **e.** Western blot analyses illustrating changes in abundance of CARM1 and PRMT1, 2, 5, and 6 in parental and CARM1 KO MCF7 and MDA-MB-231 cells. PRMT8 was not detected in either cell line and therefore not included here.

Supplementary Figure 2.

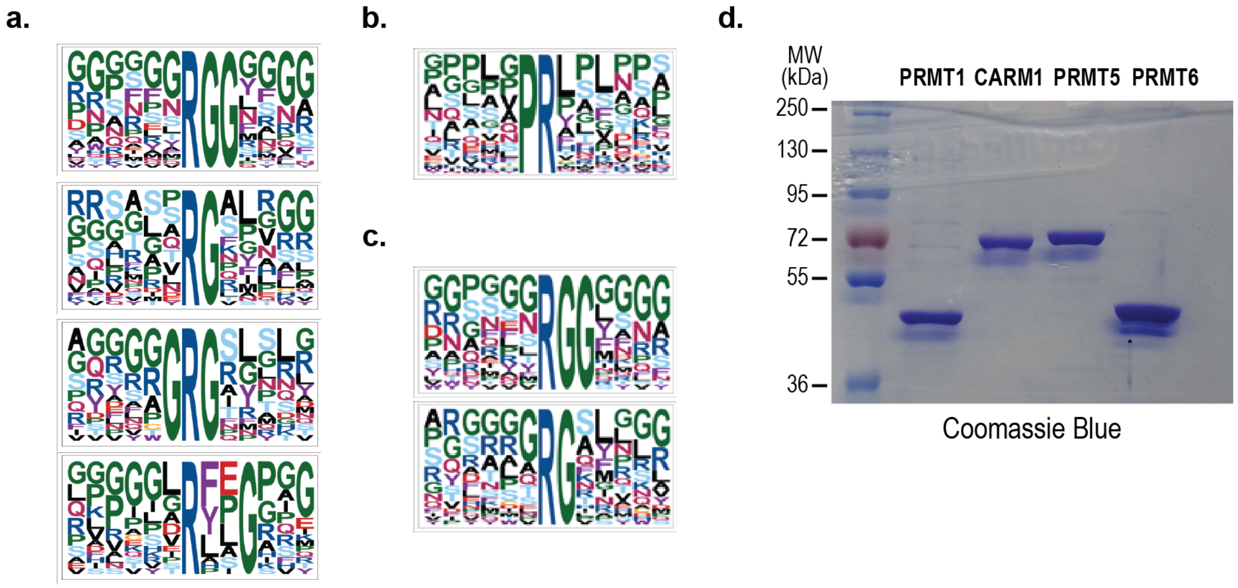

Supplementary Figure 2. Motif analyses of ADMA sites unaffected by CARM1 deletion and purification of PRMTs used in the *in vitro* methylation assays.

- **a.** Motif analyses of ADMA sites unaffected by loss of CARM1. Canonical RGG/RG and similar glycine-containing motifs were extracted from peptide sequences surrounding ADMA sites abundances of which remained unchanged in CARM1 KO cells, as compared with the parental cells.
- **b.** Motif analyses of ADMA sites encompassed by singly-methylated peptides and affected by the loss of CARM1.
- **c.** Motif analyses of ADMA sites encompassed by singly-methylated peptides and unaffected by the loss of CARM1.
- **d.** Coomassie Brilliant Blue staining of purified recombinant PRMTs from HEK293T cells used in the *in vitro* methylation assays of peptide arrays (Figure 2d and e).

Supplementary Figure 3.

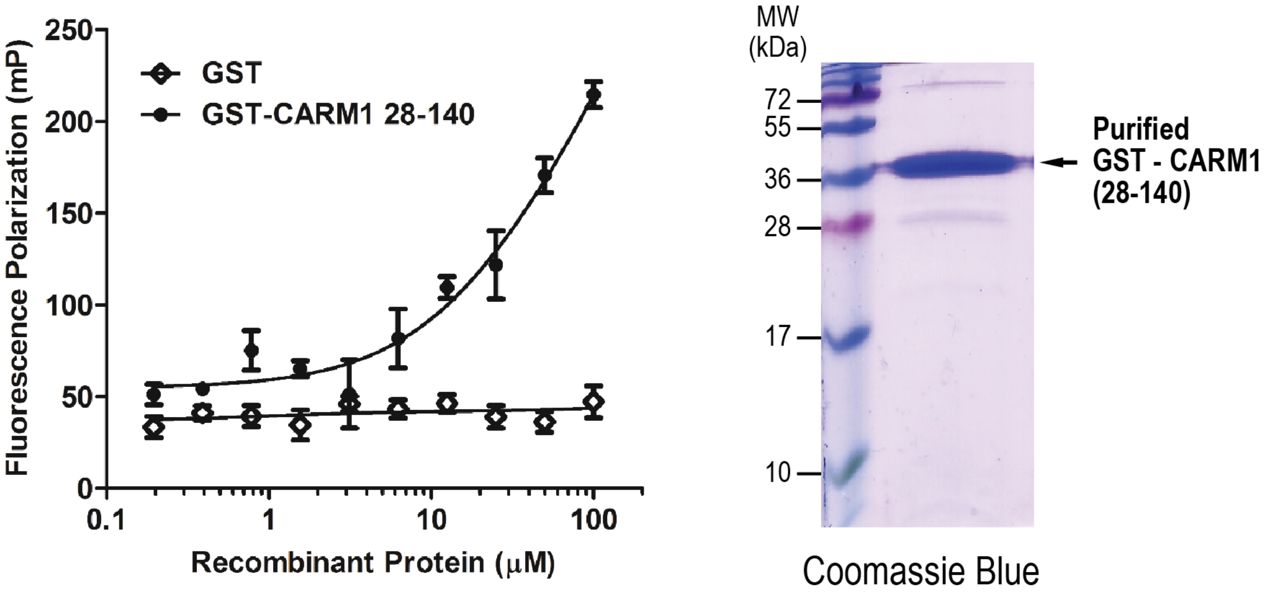

Supplementary Figure 3. Fluorescence polarization assay using purified GST-tagged N-terminal domain of CARM1 and fluorescein-labeled BAF155 peptide.

Fluorescence polarization assay using purified recombinant GST-CARM1 28-140 (*right*) and fluorescein-labeled BAF155 peptide. Pronounced increase in fluorescence polarization (*left*) was detected with the increasing concentrations of GST-CARM1 28-140, but not with the GST alone, demonstrating that the EVH1 domain of CARM1 directly interacts with the enzyme's substrate at low affinity.

Supplementary Figure 4.

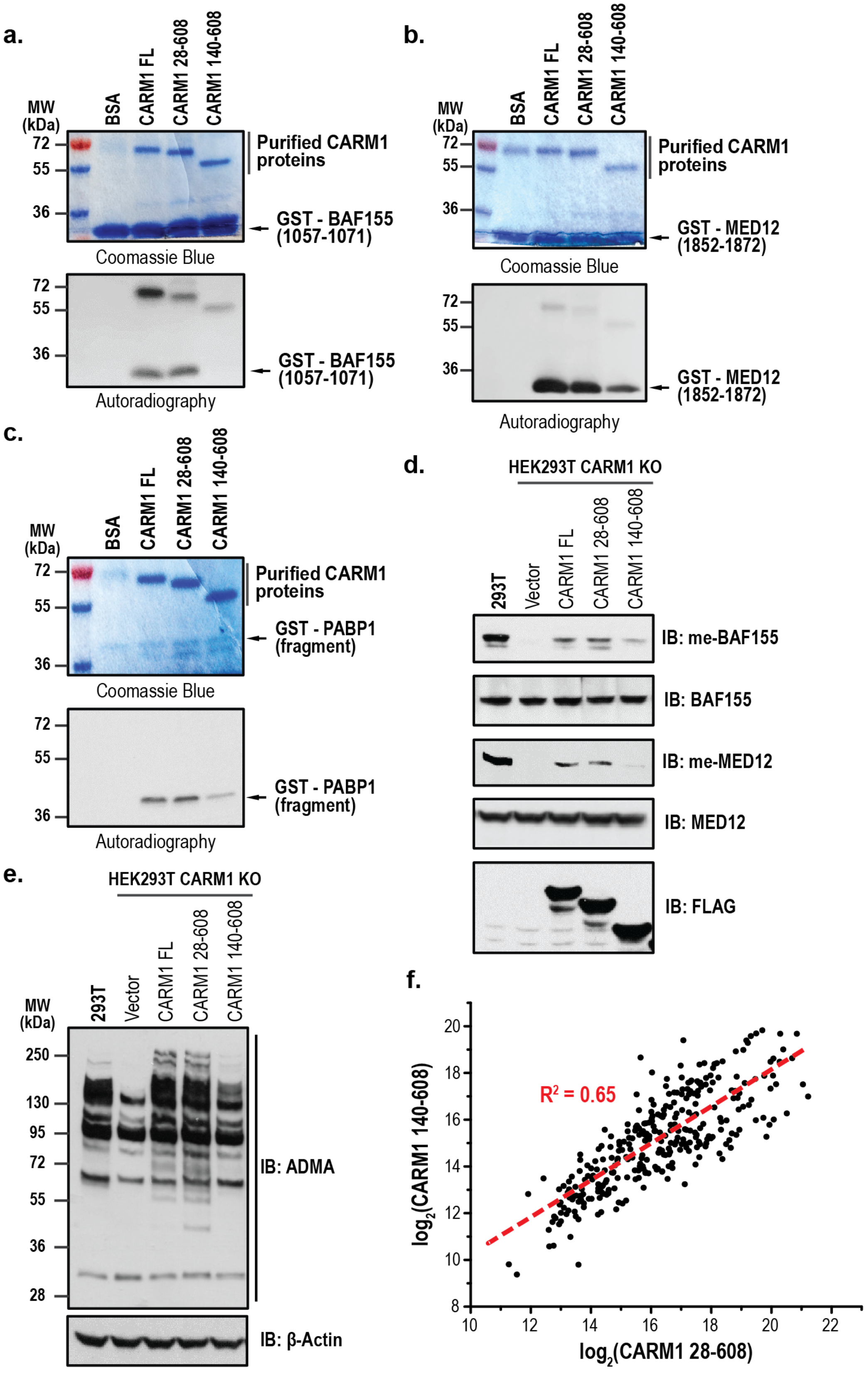

Supplementary Figure 4. Requirement of the N-terminal domain for substrate methylation by CARM1 *in vitro*.

- **a.** Coomassie Brilliant Blue staining (*top*) and autoradiograph (*bottom*) of *in vitro* methylation assays using the indicated proteins, ^{3}H-SAM, and GST-BAF155 peptide.
- **b.** Coomassie Brilliant Blue staining (*top*) and autoradiograph (*bottom*) of *in vitro* methylation assays using the indicated proteins, ^{3}H-SAM, and GST-MED12 peptide.
- **c.** Coomassie Brilliant Blue staining (*top*) and autoradiograph (*bottom*) of *in vitro* methylation assays using the indicated proteins, ^{3}H-SAM, and GST-PABP1 peptide.
- **d.** Western blot analyses of cell lysates from HEK293T cells or HEK293T CARM1 KO cells transiently transfected with the indicated FLAG-tagged CARM1 plasmids using the indicated antibodies (i.e., BAF155, me-BAF155, MED12, and me-MED12). CARM1 was detected using the anti-FLAG antibody. Reduced methylation of the known CARM1 substrates was evident in the cells expressing CARM1 140-608 construct.
- **e.** Western blot analyses of ADMA-containing proteins in total cell lysates from HEK293T cells or HEK293T CARM1 KO cells transiently transfected with the indicated plasmids. Reduction in the overall abundance of ADMA-containing proteins was observed in cells expressing CARM1 140-608. β-Actin was used as a loading control.
- **f.** Comparison of the abundance of ADMA-containing peptides in MCF7 cells expressing CARM1 28-608 and CARM1 140-608, using three biological replicas of each cell line. A loose Pearson correlation in the levels of ADMA-containing peptides was detected between two cell lines (R^2 of 0.65), indicating major differences between the ability of two CARM1 truncations to recognize and methylate its substrates.

Supplementary Figure 5.

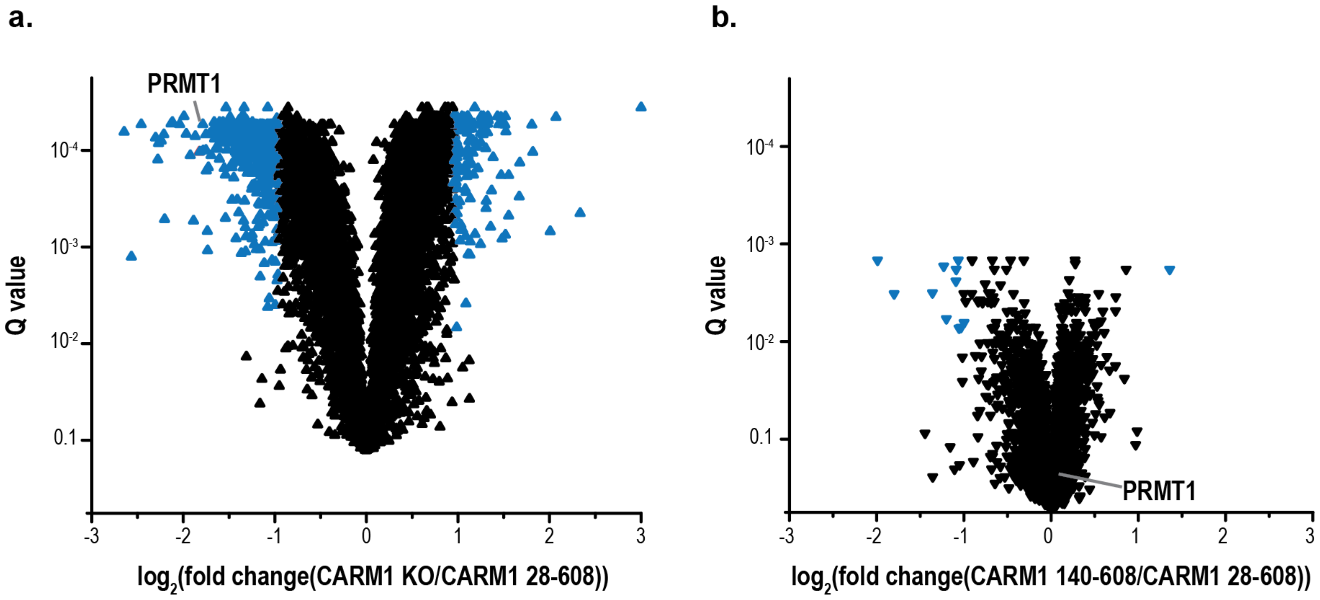

Supplementary Figure 5. Global changes in protein abundances in CARM1 KO MCF7 cells and CARM1 KO MCF7 cells stably expressing CARM1 140-608.

- **a.** A volcano plot of changes in protein abundance in CARM1 KO MCF7 cells, as compared to CARM1 KO MCF7 cells stably expressing CARM1 28-608, using three biological replicas of each cell line. The abundances of numerous proteins (in blue), including PRMT1, was reduced upon CARM1 deletion (FDR of 1%; Storey correction for multiple hypothesis testing)
- **b.** A volcano plot of changes in protein abundance in CARM1 KO MCF7 cells stably expressing CARM1 140-608, as compared to CARM1 KO MCF7 cells stably expressing CARM1 28-608, using three biological replicas of each cell line. The abundances of only a few proteins (in blue) were significantly affected by the truncation of the EVH1 domain (FDR of 1%; Storey correction for multiple hypothesis testing). PRMT1 abundance was not changed in this cell line.

Supplementary Figure 6.

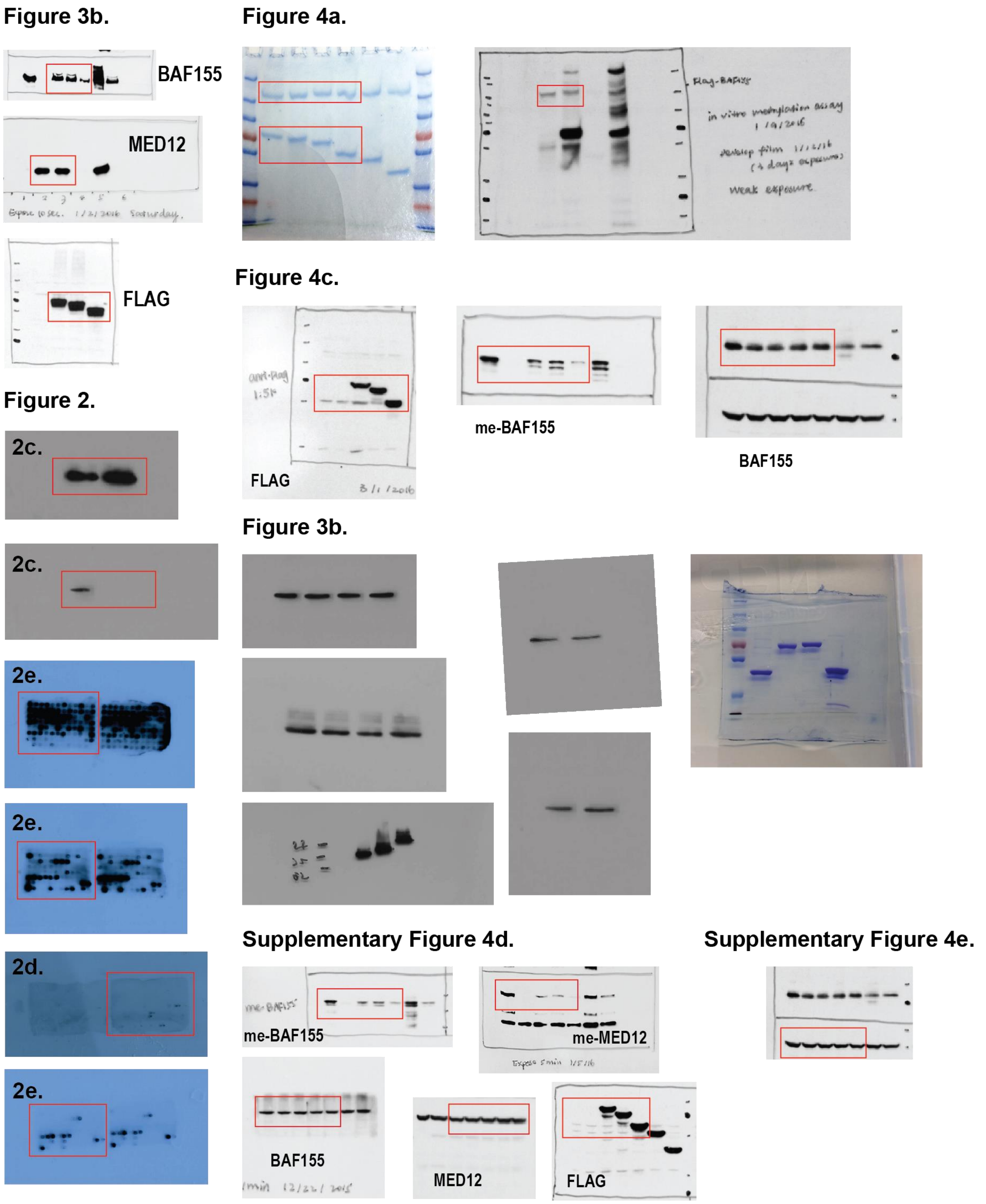

Supplementary Figure 6. Uncropped images of blots and gels. The red frame indicates the position of cropping. The respective figure numbers are provided.

Supplementary Table 1. Oncogenic substrates of CARM1.

Protein	Methylation site(s)	Proposed role in cancer
ARID1A	R391, 429, 557	Transcription regulator via chromatin organization; proposed tumor suppressor frequently lost or mutated in human cancers
ARID1B	R557	Transcription regulator via chromatin rearrangement, essential for survival of ARID1A-deficient cancers
BCL11B	R322	Tumor-suppressor protein involved in T-cell lymphomas
CDK12	R1407	Transcriptional activator of DNA damage response factors
MLL3	R2434, 2444, 2454, 2571, 4196, 4220, 4202	Histone methyltransferase involved in transcriptional coactivation
MLL2	R2431, 2804, 2833, 2906, 2908, 3730	Histone methyltransferase; a driver in numerous cancer types that causes genome instability
MAP2K4	R69	Protein kinase that promotes tumor metastasis in prostate cancer via target phosphorylation and may function as a tumor suppressor in other cancers
MED12	R1854, 1859, 1862, 1899, 1910, 1912	Component of the mediator complex that controls response to multiple cancer drugs through regulation of TGF-β receptor signaling
MLLT6	R589	Proto-oncogene; chromosomal aberrations involving MLLT6 is associated with acute leukemias
NCOA3	R1171, 1177, 1188	Gene expression co-activator aberrantly expressed in several cancers
NCOR2	R1661, 1679	Mouse insertional mutagenesis experiments support NCOR2 as a cancer causing gene
PML	R599	Regulator of DNA repair, alternative lengthening of telomeres, transcriptional control, apoptosis, and senescence
TET2	R1682	Methylcytosine dioxygenase with a prominent role in DNA demethylation; its loss promotes prostate cancer and blood cancers
TFE3	R188	Potent transcription activator that forms fusion products with other proteins with variable preservation of the CARM1 methylation site
TPR	R2163	Component of protein trafficking complex; its N-terminus is involved in activation of oncogenic kinases
TRIM24	R539, 548	Transcriptional coactivator that interacts with numerous nuclear receptors and coactivators and modulates the transcription of target genes; oncogene in prostate cancer
TRIM33	R440, 515, 555, 558, 568, 598	Negative regulator of several transcriptional complexes through histone modification and binding

Supplementary Table 2. Cellular pathways enriched among putative CARM1 substrates.

Cellular pathway (Reactome 2016)	Combined score (Enrichr)	Gene names
Gene expression	29.8	SF3B2; TAF9; ADAR; SMG7; ELAVL1; PSMA8; MED12; PPP1R13L; PCF11; TPR; ZNF703; SMN1; TAF9B; EXOSC1; WDR36; SF3A1; NCOA6; TET2; CD3EAP; RPRD2; PML; PATL1; RUNX2; NCOR2; AIMP2; HNRNPM; CNOT2; XRN2; PABPC1; CDK12; MAML3; DCP1A; TAF4; TRIM33
Chromatin organization	12.9	NCOR2; KMT2D; KMT2C; TAF9; GPS2; ARID1A; ARID1B; CLOCK; HCFC1
Chromatin modifying enzymes	12.8	NCOR2; KMT2D; KMT2C; TAF9; GPS2; ARID1A; ARID1B; CLOCK; HCFC1
mRNA decay	11.4	CNOT2; PABPC1; DCP1A; EXOSC1; PATL1
Regulation of lipid metabolism by PPARα	9.1	NCOR2; MED12; CPT2; NCOA6; NCOA3; CLOCK
Regulation of mRNA stability	8.2	PABPC1; DCP1A; ELAVL1 EXOSC1; PSMA8
Generic transcription pathway	6.0	NCOA6; TAF9; RUNX2; PML; MED12; NCOR2; PPP1R13L; CNOT2; ZNF703; CDK12; MAML3; TAF4; TAF9B; TRIM33
Transcriptional regulation of white adipocyte differentiation	5.2	NCOR2; MED12; NCOA6; NCOA3
mRNA splicing	5.2	HNRNPM; SF3B2; SF3A1; PCF11; ELAVL1
Activation of HOX genes during differentiation	5.0	KMT2D; NCOA6; NCOA3; KMT2C
Regulation of TP53 Activity	4.9	PPP1R13L; TAF9; TAF9B; TAF4; PML

Supplementary Table 3. Motifs in the vicinity of ADMA sites regulated by CARM1.

Motif	Matches in the dataset	% Dataset	Fold increase over general frequency in the human proteome
**PR**	86	28.8	5.1
**RxxxP**	58	19.4	4.9
**RxP**	38	12.7	5.1
**PRxxxxP**	34	11.4	15.1

Supplementary Table 4. Motifs in the vicinity of ADMA sites regulated by other PRMTs.

Motif	Matches in the dataset	% Dataset	Fold increase over general frequency in the human proteome
**RG**	47	29	4.1
**RGG**	34	21	32.4
**GRG**	20	12.3	22.6
**PRxxxxP**	20	12.3	3.6

Supplementary Table 5. Experimental setups and used TMT labels.

Experiment	Biological replicate	TMT channel
**#1** (6-plex): wild type (WT) and CARM1 knockout (KO) MCF7 cells	WT1	126C
	WT2	127C
	WT3	128C
	KO1	129C
	KO2	130C
	KO3	131N
**#2** (6-plex): wild type (WT) and CARM1 knockout (KO) MDA-MB-231 cells	WT1	126C
	WT2	127N
	WT3	127C
	KO1	128N
	KO2	128C
	KO3	129N
**#3** (9-plex): knock-in wild type CARM1 (WT), knock-in CARM1 140-608 (TR), and CARM1 knockout (KO) MCF7 cells	WT1	126C
	WT2	127N
	WT3	127C
	TR1	128C
	TR2	129N
	TR3	129C
	KO1	130N
	KO2	130C
	KO3	131N
